# Minimally invasive operation of severe complex thoracic deformity with Wenlin procedure and Wung procedure

**DOI:** 10.1093/jscr/rjac473

**Published:** 2022-10-25

**Authors:** Wenlin Wang, Weiguang Long, Yang Liu, Bin Cai

**Affiliations:** Department of Chest Wall Surgery, Guangdong Second Provincial General Hospital, Guangzhou, China; Department of Chest Wall Surgery, Guangdong Second Provincial General Hospital, Guangzhou, China; Department of Chest Wall Surgery, Guangdong Second Provincial General Hospital, Guangzhou, China; Department of Chest Wall Surgery, Guangdong Second Provincial General Hospital, Guangzhou, China

## Abstract

Severe complex thoracic deformity has both protrusion and depression on the chest wall. General minimally invasive operation cannot complete the treatment. We recently treated a 15-year-old boy with Wenlin procedure and Wung procedure, and achieved satisfactory results.

## INTRODUCTION

Thoracic deformity is one of the surgical diseases of the chest wall [1]. The most common deformity is simple depression or protrusion on the chest wall [2, 3]. However, in clinic, there are often deformities combined with both depression and protrusion, which are complex thoracic deformities [4, 5]. Their treatments are extremely challenging, always requiring special techniques.

## CASE REPORT

**Figure 1:**
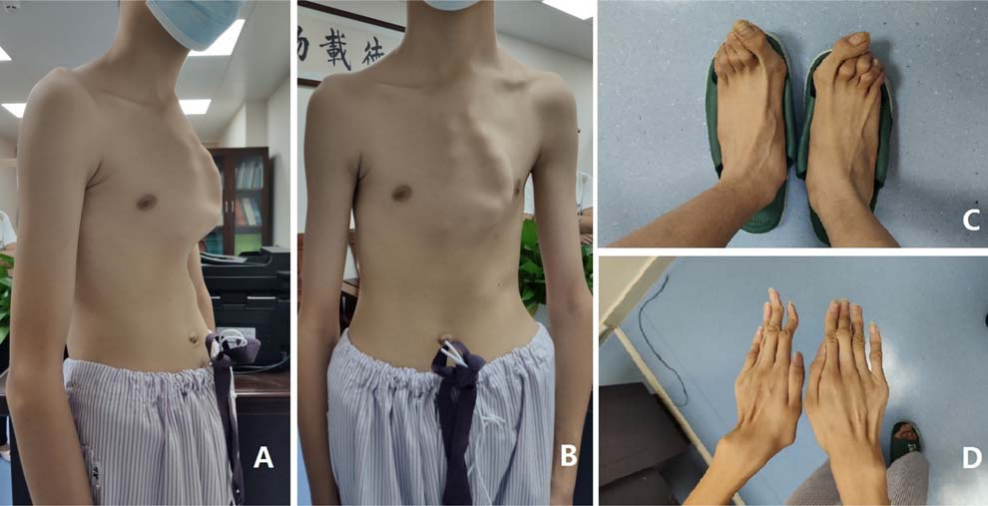
**(A)** and **(B)** appearance of chest wall before operation, **(C)** feet and **(D)** hands.

**Figure 2:**
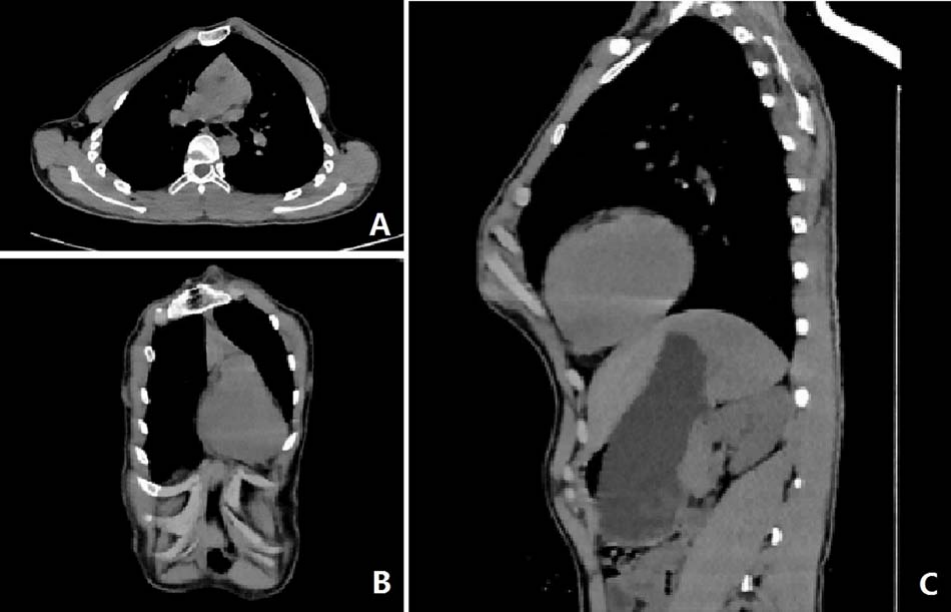
CT examination images before operation.

**Figure 3:**
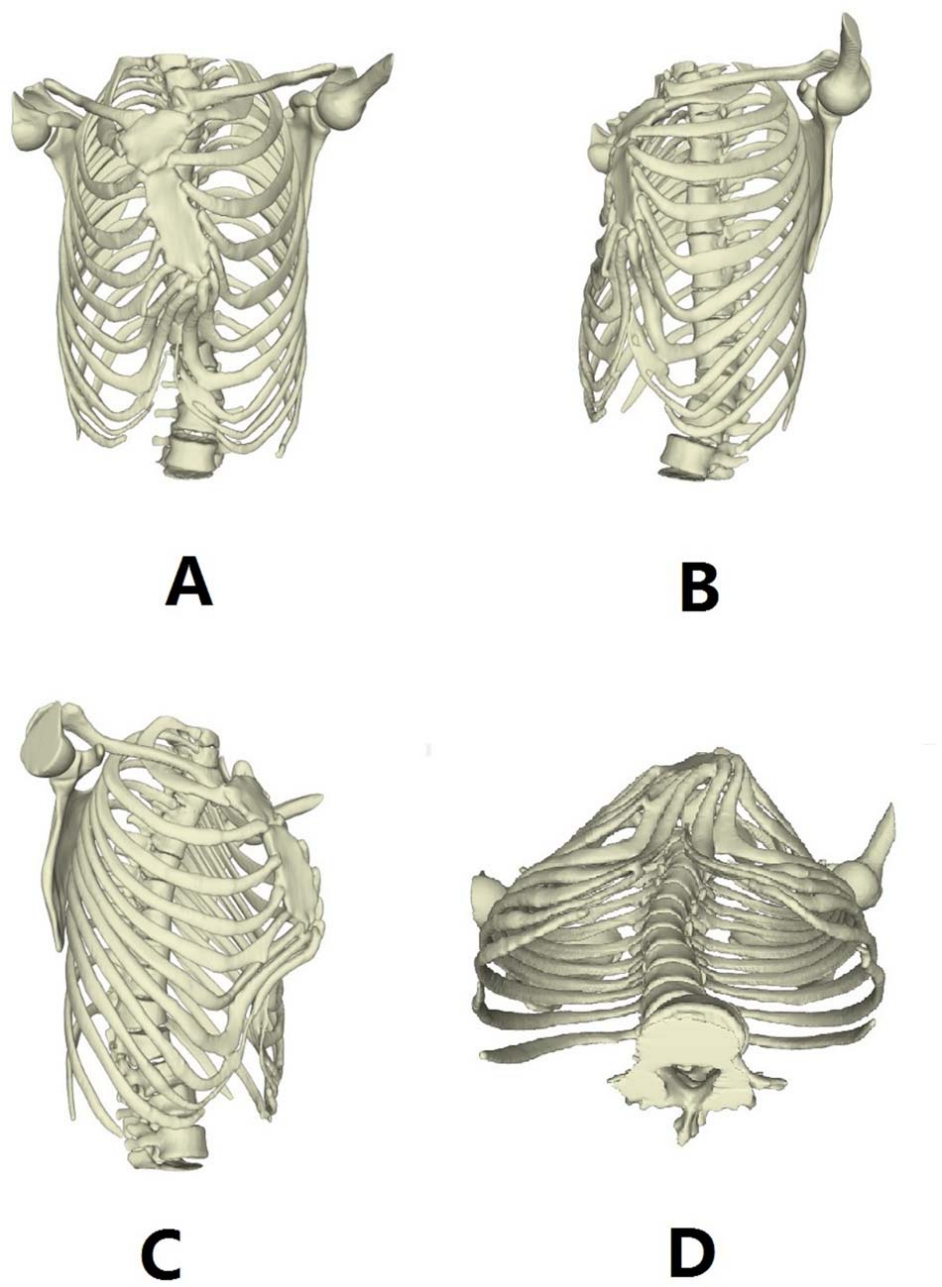
3D reconstruction images of thorax before operation.

Our patient was a 15-year-old boy. He was found to have abnormal chest wall appearance at early age. The early deformity was mild but began to worsen at the age of 10 years, mainly manifested as protrusion of the anterior chest wall. He had no discomfort, but was dissatisfied with the appearance. In order to treat the deformity, he was admitted to our hospital recently for surgery. Preoperative physical examination showed that the anterior chest wall was protrusive, but there were localized depressions in the middle and below part of the chest wall (Fig. 1A, B). He also had obvious deformities of toes and fingers (Fig. 1C,D). Preoperative imaging examination revealed irregular protrusion of the anterior chest wall with localized depressions; severe distortion of sternum, costal cartilages and ribs; and mild scoliosis of spine (Figs 2 and 3). The operation was performed under general anesthesia.

**Figure 4:**
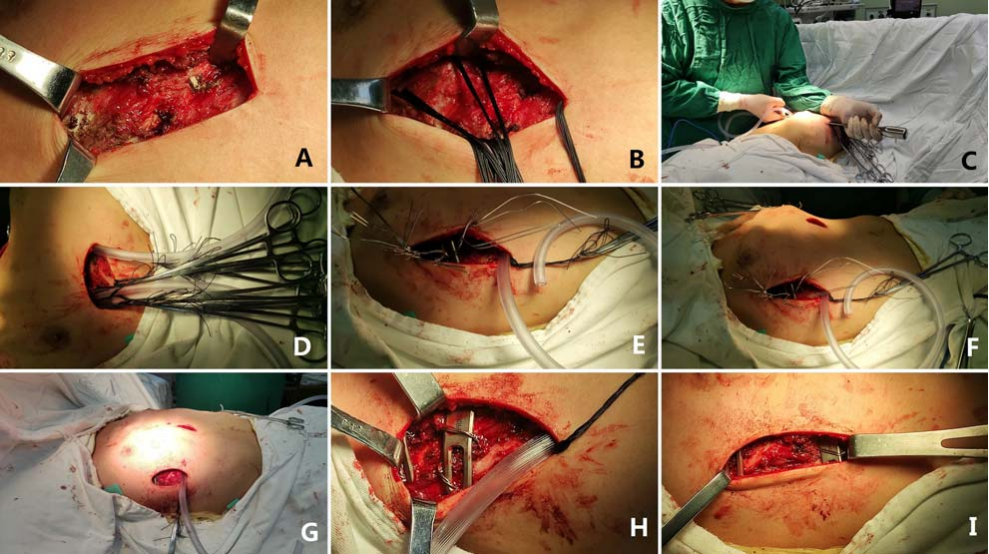
**(A)**, **(B)**, **(C)**, **(D)**, **(E)**, **(F)**, **(G)** and **(H)** surgical field of Wenlin procedure; and **(I)** surgical field after Wung procedure.

**Figure 5:**
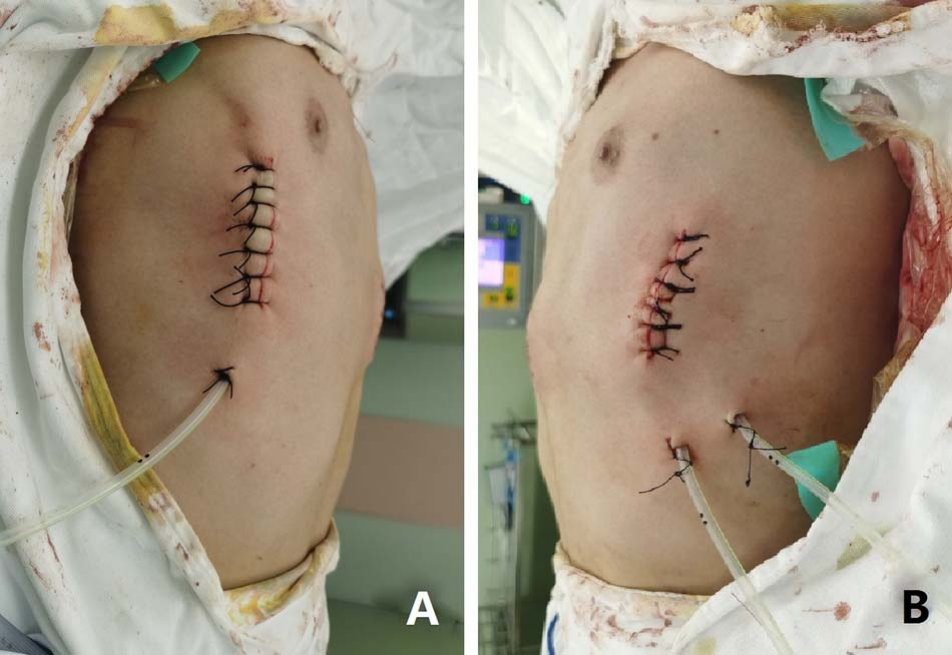
Appearance of chest wall after operation.

**Figure 6:**
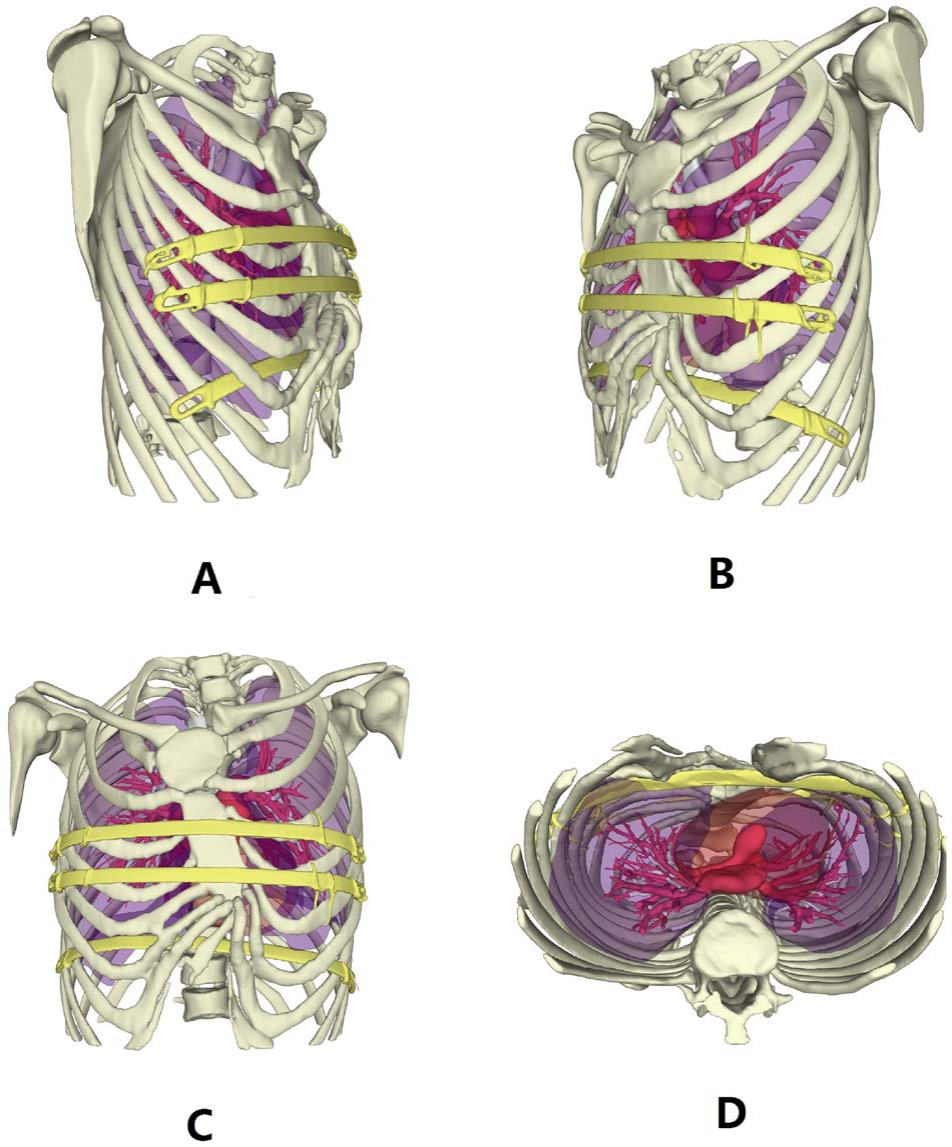
3D reconstruction images of thorax after operation.

Three skin incisions were made on both sides of the chest wall and in front of the xiphoid process. Wenlin procedure was completed with two steel bars to flatten the protrusion [3–5], while Wung procedure was performed with the third steel bar to support the depression [4–6](Fig. 4). During the operation, all the steel bars were fixed with Wang techniques [7]. After the bars were firmly fixed, the operation was completed and the deformity was totally corrected (Fig. 5). No complications occurred during the operation. Postoperative imaging examination showed that the positions of the steel bars were satisfactory and the thoracic shape was basically normal (Fig. 6). The patient was discharged 1 week after operation.

## DISCUSSION

The most common thoracic deformity is pectus excavatum, followed by pectus carinatum [2, 3]. These deformities are simple depression or protrusion, and their treatments are not complicated, but there are often deformities including both depression and protrusion simultaneously. These deformities are complex deformities, the treatment of which is relatively difficult [4].

Up to now, there are roughly the following operations for the treatment of simple depression deformities, namely Nuss procedure [2], Wang procedure [2] and Wung procedure [6]. The operations for the treatment of simple protrusion deformities include Abramson procedure [8] and Wenlin procedure [3]. Because there are both protrusion and depression in the complex deformity, it can be considered to use a combination of the two procedures mentioned above at the same time. In the past, Nuss procedure and Abramson procedure were used to treat complex deformities, which were called Sandwich technique [9]. Since both procedures have various disadvantages, the combination is not an ideal choice. After a long time practice, we found that the combination of Wenlin procedure and Wung procedure was more suitable for the operation of such complex deformities, especially for the severe cases [4, 5]. One of the biggest features of this combination is the use of Wang technique for steel bar fixation [7]. Since the short fixation plates are no longer used, the operation will be simpler, and the incision may be greatly shortened. In addition, due to the special technology of placing steel bar in the Wung procedure, the safety of the operation will be greatly improved [6].

In the past, we have used this combination technology to treat many complex deformities [4, 5]. This patient is rare, and the most serious of our patients. The operative success further proves the effectiveness of our method.

## CONFLICT OF INTEREST STATEMENT

None declared.
